# Telephone-based aftercare groups for family carers of people with dementia: study protocol of the Talking Time – REHAB project

**DOI:** 10.1186/s12913-019-4003-7

**Published:** 2019-03-21

**Authors:** Martin Berwig, Susanne Lessing, Ruth Deck

**Affiliations:** 1 Clinic for Cognitive Neurology, Medical Faculty, University of Leipzig, Leipzig, Germany; 2 Institute for Social Medicine and Epidemiology, University of Lübeck, Lübeck, Germany; 3 Rehabilitation Clinic for Family Carers, AMEOS Rehab Clinical Centre Ratzeburg, Ratzeburg, Germany

**Keywords:** Dementia, Family carers, Telephone-based, Aftercare groups, Psycho-social intervention, Social support, Participation

## Abstract

**Background:**

More than one million people in Germany live with dementia. Most of these people are cared for at home in the family setting. Supporting and caring for people with dementia is time-consuming, and family carers often have high stress levels and are at an increased risk of becoming physically and mentally ill. Medical rehabilitation (rehab) helps to relieve family carers and provide them with strategies to cope with stress. The aim of this study is to improve the sustainability of a multimodal rehab program for family carers of people with dementia. Research question: can the effects of this rehab be maintained through telephone-based aftercare groups following the rehab program?

**Methods:**

A prospective randomized controlled longitudinal trial is performed. The intervention group (IG) participates in telephone-based aftercare groups; the control group (CG) receives treatment as usual. For evaluation, a mixed-methods approach is used. The effects of the intervention are quantitatively evaluated by written questionnaires at four measuring points (pre- and post-rehab, as well as 6 and 12 months after the end of rehab). Primary outcome: participation (IMET). Secondary outcomes: Depressive Mood State CES-D, General Complaints SCL-90-R, Subjective Quality of Life WHOQUOL-BREF, Social Support F-SozU, performance in different areas of life, single scales, and support offers (single items). The intervention process is evaluated through qualitative interviews and focus groups with regard to the acceptance of and satisfaction with the aftercare offered; in addition, a health economic evaluation is performed using the EQ-5D questionnaire. Rehabilitants are included in the study (*N* = 103 each in the IG and CG) who, accompanied by their family members with dementia, participate in the rehab measure in Ratzeburg. The IG participates monthly in 6 telephone aftercare groups over a period of 6 months. Typical stress situations are discussed and worked on.

**Discussion:**

Upon successful evaluation, the offer to participate in telephone-based aftercare groups can be firmly established in the participating rehab clinic. Through minor adjustments, the offer would also be suitable for carers of physically ill people and for non-nursing-specific rehabilitation indications.

**Trial registration:**

German Clinical Trials Register: DRKS00013736, May 14, 2018.

## Background

Currently, in Germany, there are 1–1.5 million people living with dementia, and it is expected that this number will increase to 3.0 million people by 2050 [1]. Worldwide, and particularly in Germany, the family remains the cornerstone of caring for the elderly [2], and carers, therefore, bear a large part of the responsibility for caring for and supporting people with dementia. More than two-thirds (70%) of people in need of care and who live at home are primarily cared for by partners and children [3]. Approximately half of those in need of care are people with dementia [4].

Supporting and caring for people with dementia is time-consuming and requires significant personal commitment and day-to-day management. Therefore, family carers of people with dementia often have higher stress levels than carers of the physically frail elderly [5, 6], and they are at an increased risk of becoming physically and mentally ill [5]. The development of the stress experience of family carers is complex [7]; the obviously non-cognitive symptoms of dementia, such as psychotic symptoms, depression and challenging behaviour, are of particular importance [8, 9]. Challenging behaviour refers to recurring behaviours that are perceived by the social environment as maladaptive and inappropriate to the situation (e.g., aggressiveness, irritability, anxiety).

To date, many psychosocial interventions have been developed that aim to improve the mental and physical health of family carers and reduce their stress experience [5]. These interventions vary in format (individual or group format) and content (psychoeducation, symptom assessment, problem-solving, skills training, stress management strategies, behavioural modification). A meta-analysis [10] concludes that individualized and structured multicomponent interventions are most effective. Against this background, particularly for carers of people with dementia, a rehabilitation (rehab) concept has been designed at a rehab centre in Schleswig-Holstein: family carers receive specific offerings that help them build up their strength resources, and they are trained to better meet the demands of caring for their relatives with dementia in their everyday lives. This program should enable improved participation in life and in the community. To facilitate access to the rehab, the dementia-affected family member can also be accommodated and supported in spatial proximity. Thus, on the one hand, the concept enables preservation of the contact opportunity between the carer and his family member with dementia; on the other hand, it allows the rehabilitants to concentrate fully on their therapies.

The rehabilitants (family carers) receive comprehensive, multi-professional therapeutic treatments, as well as other forms of relief, including individual and group psychotherapeutic sessions, medical visits, nursing training, ergo-, art-, music- and physiotherapy. Furthermore, the rehabilitants participate in autogenic training, progressive muscle relaxation and dementia-specific family carers training, as well as social counselling. The concept has been implemented since May 1, 2012 and, thus far, it is unique in Germany in this form. Funding for such a rehab project, at which the relatives can be cared for and brought along, has been assured by the German nursing reorientation law since January 2013. The costs for the person with dementia are covered by the health insurance of the rehabilitant. If there is a need for care, the nursing care insurance of the family member with dementia will proportionally take over the costs of the corresponding short-term or preventive care.

The current results of an observational study to evaluate the rehab program in Ratzeburg show clear and statistically significant changes regarding depression and general complaints, as well as the global quality of life of family carers at the end of rehab [11]. However, these effects were reduced to nearly baseline levels at the 6-month follow-up. Although these results confirm the basic efficacy of medical rehab in most indications after the end of the measure [12], they also show, like other studies in this field [13, 14], that these effects are not sustainable.

A time-limited rehab of usually 3 weeks is not sufficient in most cases to persistently stabilize the treatment’s success. Rehabilitation aftercare is a possibility to support the transfer of the learned contents to everyday life. Meanwhile, there are numerous aftercare programs that, based on the specific problem situations of rehabilitants, facilitate the transition from rehab to everyday life. Usually, they take place nearby at home at contracted outpatient facilities of the payers. However, against the background of the nursing and caring for the partner with dementia, utilization of usual aftercare programs is difficult to implement for family carers. This point is the start of the study project *Talking Time – REHAB as Aftercare*. It aims to support the transfer of what has been learned in rehab to the home environment through location-independent telephone-based aftercare groups, thereby consolidating the rehab effects. Telephone aftercare has been proven in practice. The 16 randomized, controlled trials found in a literature review suggest that telephone-based aftercare can increase the sustainability of rehab [15].

However, a wide variance in the underlying concepts was determined in the included studies. The strongest effects were obtained by concepts with the highest care intensity. Most of the included studies involved dyadic concepts. In contrast to existing studies, in Talking Time – REHAB, a group setting was selected for the telephone aftercare. With telephone-based aftercare in a small, professionally moderated group, a higher intensity of care can be assumed; therefore, a large sustainability effect can be expected. As part of the TALKING TIME project [16], for the first time in Germany, structured telephone-based support groups for carers of people with dementia were implemented. Talking Time – REHAB is formally based on the structure of the TALKING TIME intervention; however, the content is adapted to ensure the function of rehab aftercare.

### Study aim and research question

For the effect evaluation of this study project, the following primary questions and/or hypotheses arise:

1. Does participation in the telephone-based aftercare groups lead to increased social participation by family carers of people with dementia?
2. Can the health-related effects and competences obtained in multimodal rehab be maintained or even increased after rehab by participating in telephone-based aftercare groups?

The process evaluation of Talking Time – REHAB focuses on the following questions:

1. What structures and processes contribute to the successful implementation of the aftercare offering?
2. What promoting factors or hindrances influence successful implementation?
3. How satisfied are the participants with the individual aspects of the aftercare offering?

## Methods

### Design

**Fig. 1:**
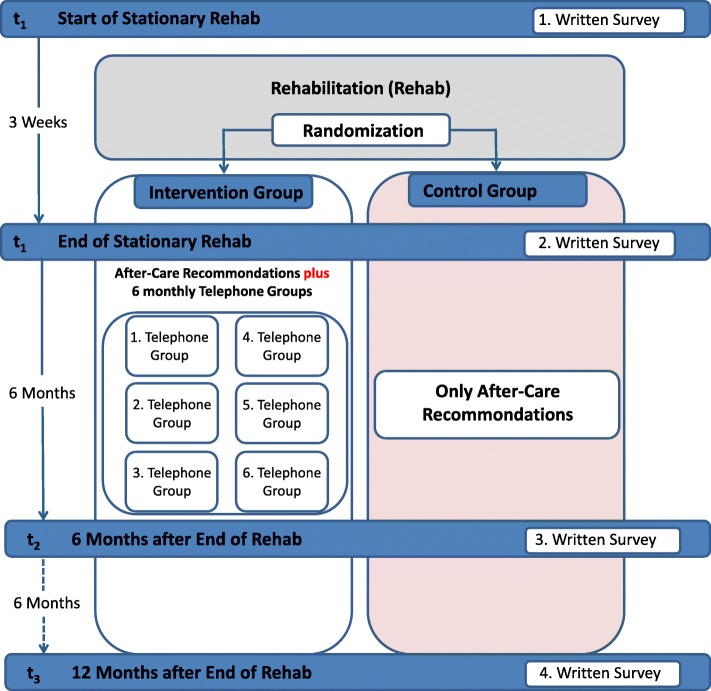
Study design

To investigate the long-term effects of telephone-based aftercare groups, a randomized controlled prospective longitudinal study is being conducted at one rehab centre. For the effect and process evaluation, in the sense of a mixed-method approach, both quantitative and qualitative methods are used [17]. Rehabilitants receive specific rehab for family carers of people with dementia and are randomized at the end of the measure. The randomization is performed externally by the Institute for Social Medicine and Epidemiology (ISE) of the University of Lübeck by generating a randomization list with the statistics program SPSS 22.0. After the rehab, at six appointments and in addition to the aftercare recommendations by the social worker, the intervention group receives telephone-based aftercare in groups, and the control group receives only the aftercare recommendations. The study design includes four measurement times: at the beginning (t0), at the end of the rehab (t1), 6 months after the end of the rehab (for the IG after completion of telephone-based aftercare groups) (t2) and 12 months after rehab ends (t3). Figure 1 represents the study flow.

### Sample size calculation

As no studies in the field of nursing care have investigated the effects of aftercare in addition to a supportive intervention for carers, the sample size calculation for this study is based on the current results of the evaluation study on the effectiveness of the rehab program for family carers of people with dementia without aftercare (“standard care”), upon which this study project is based [11], and the results of a 2012 study of the impact of individualized aftercare on long-term rehab effects [18].

In the study with family carers, six months after rehab ended, the outcome parameter of participation, which was captured with the Index for the Measurement of Restrictions on Participation (Index zur Messung von Einschränkungen der Teilhabe, IMET [19]), showed that the rehab effect had nearly completely disappeared (ES = 0.06).

In the rehab aftercare study for the indication lower back pain, the same effect was observed for participation (IMET) in the CG [18], while the rehabilitants of the IG with individualized aftercare still showed an approximately medium effect size after 12 months (ES = 0.44).

Due to the consistently strong effects at the end of rehab in most recorded outcomes in the evaluation study for the rehab program in Ratzeburg, we expect for this controlled study, with telephone-based aftercare at the 6-month follow-up, an average effect of ES = 0.5 in the IG and a small effect of ES = 0.06 in the CG. Using a test power of 80% as the base, a two-sided t-test with an alpha error level of 5% requires a net group size of *N* = 82 each for the IG and CG. Based on experience with the current evaluation study of the rehab program in Ratzeburg and due to the high degree of loyalty of the rehabilitants to the rehab facility, we assume a moderate dropout rate of 20%. To evaluate *N* = 82 rehabilitants per group, initially, 103 participants per study group should be included. The rehab centre treats between 210 and 330 carers per year. The required number of cases can thus be achieved within the envisaged recruitment time.

### Recruitment of rehabilitants

All rehabilitants who in the course of a year, accompanied by a relative with dementia, consecutive newly admitted to AMEOS Rehab Clinical Centre Ratzeburg, a rehab clinic for family carers, will be addressed as potential study participants. Exclusion criteria are the presence of a personality disorder, psychotic symptoms, language barriers and cognitive impairments. The rehabilitants are first informed verbally about the study during an information event and asked to participate. Written information material will be handed out, and for those willing to participate, a written informed consent will be obtained.

### Intervention

#### Intervention group

The primary focus of the present study is the implementation of telephone-based aftercare, which is conducted in the form of aftercare groups. The structure of these groups is based on the support groups, as implemented by telephone in the TALKING TIME project [16]. The theoretical framework of these telephone rehab aftercare groups is provided using three approaches. 1.) According to Ruth Cohn’s theme-centred interaction (TCI) model [20], interaction and communication in a group are influenced by the factual, ego, and we-level. The factual level includes the theme of the telephone-based aftercare group to be discussed; the ego level focuses on the individual needs and current emotional state of each group participant; the we-level encompasses the group dynamic processes. To enable effective group work, these three levels must be in balance. Therefore, the role of the moderator is to keep these three levels in balance. This goal is completed by the moderator supporting the self-regulation of the group, strengthening the autonomy of the group members and creating an open atmosphere for communication and interaction. 2.) Based on systemic therapy [21], psychological burdens are considered not only at the individual level, but also, the systems of interpersonal relationships in which a person with dementia lives take centre place. The objective is to change these relationships and the interaction in different systems. In the context of people with dementia, this view can be briefly characterized by the concept of ‘dementia as a family disease’. Considered are the role changes, acceptance of new functions and changes in the relationship in general, the gradual farewell from a close relative, and the need to increasingly turn to that person because of the growing dependency. Also touched upon is the issue of ‘ambiguous loss’, that is, the person with dementia is still physically there but increasingly mentally absent [22]. 3.) From the broad spectrum of behavioural therapy, which understands psychological burdens as conditioned by learning experiences and as being changeable [23], the following principles take effect: a structured and transparent procedure, clear group structures, an emotion-, problem- and resource-oriented approach and support for self-help [21]. From the behavioural and systemic approaches, strategies and competences can be derived, such as how the family carer can better deal with ‘challenging behaviours’ in domestic everyday life. Communicating these skills is of particular importance when the aftercare recommendations target these challenging behaviours in everyday life.

The temporal and organizational procedure of the Talking Time – REHAB aftercare measurement consists of the following four components.

1. **At the end of rehab – inclusion and preparation.** At the end of the rehab stay, a social worker, who is familiar with German social security statute book XI (Sozialgesetzbuch, SGB XI) and responsible for the moderation of the aftercare groups, compiles a portfolio of aftercare recommendations that are adapted to the individual situation of the respective family carer. These recommendations largely refer to possible strategies, as well as the individual situation of the carer, offering meaningful support but also providing recommendations for self-care in daily life, including nursing and supporting. The aftercare recommendations are discussed with the family caregivers at the end of rehab. In this discussion, the appointments of the telephone-based aftercare groups will be determined, and conversational rules for the groups are discussed. The rehabilitant receives a written checklist with all important information, including the conversation rules and the group folder (see below), to take home.
2. **Reminder call**. The moderator calls each rehabilitant of the aftercare group 1 day before the next scheduled appointment. Such a reminder call lasts approximately 3–5 min and is necessary because, with a one-month interval of the aftercare group, the next appointment may be lost or forgotten by the participants.
3. **Structured telephone-based aftercare groups**: Overall, the intervention provides for participation in six telephone-based aftercare group sessions within 6 months (every 4 weeks), each lasting approximately 60 min. The groups consist of a maximum of five caregivers and a moderator, who leads the group, and the groups are closed; the participants in a telephone-based aftercare group remain constant over the period of the six appointments. These groups are conducted as dial-out telephone conferences using a telephone computer system. The participants and the moderator are simultaneous automatically called by the telephone computer system and are connected to one another in the virtual conference room, after they have confirmed their personal receipt of the call by pressing the ‘1’ key of the dial pad of their telephone. In this manner, the telephone computer system confirms that an answering machine has not accepted the call. At the beginning of each group discussion, each participant gives a short update on his individual care and support situation and the status of the implementation of his aftercare recommendations. Afterwards, in all sessions, except for the first one, one of five themes will be introduced by the moderator: 1.) aftercare, implementation of the aftercare recommendations developed during the rehab; 2.) dealing with the relative with dementia; 3.) self-care (What am I doing for myself? When and where can I draw strength?); 4.) ambiguous loss, coping with grief and loss in dementia; and 5. social networks, perception and use of networks. To support the thematic introduction of each support group, each participant will receive a group folder developed particularly for the Talking Time – REHAB study, summarizing information on the themes of the Talking Time – REHAB sessions and including a checklist regarding technical issues (e.g., ‘what is the battery status of my telephone?’), which must be considered prior to each group session. The group folder can also be used as a workbook (e.g., for notes) during the support group meeting. This initial round, including impulse presentation, should take approximately 10–15 min. The remaining 45 to 50 min are available for the moderated exchange of experiences and the discussion between family carers. At the end of the group discussion, the moderator summarizes the content of each session. For quality assurance, the moderator is supervised by a psychological psychotherapist after the third and sixth session of the respective aftercare group.
4. **Follow-up work of telephone-based aftercare groups**: Each group discussion is evaluated by each participant in writing. Before leaving the rehab facility, the participants receive a structured evaluation form for each aftercare group appointment. Using this form, family carers are encouraged to think about and evaluate each telephone group session (e.g., regarding the relevance of the topic of the session, open questions, helpfulness of the meeting, moderation of the group, well-being in the group or implementation of aftercare recommendations, etc.) The form is sent back to the moderator immediately after the telephone group session and provides the basis for the preparation of the next telephone group, but can also help in the sense of a formative process evaluation (see below, section on ‘process evaluation’) to optimize the behaviour of the moderator in the group situation. At the end of the intervention, as part of the summative process evaluation, all questionnaires will be sent to the ISE.

#### Control group

The family caregivers of the control group will receive only a portfolio of aftercare recommendations that are adapted to the individual situation of the respective family carer at the end of rehab. This recommendation will be self-implemented and is not accompanied by any aftercare measurement. Between T1 and T2, they will continue with their usual activities or on-site services at home, without restriction (usual care).

### Effect evaluation

To evaluate the intervention effects, quantitative measurements will be applied. The measurements were chosen based on their appropriateness for the scientific issues, the target intervention, sample, data collection procedure (written survey) and psychometric properties. Table 1 summarizes all core measurement instruments to be used in this study.

**Table 1:** Core set of used instruments

Dimensions	Measurement Instrument	t_0_	t_1_	t_3_	t_4_
*Primary Outcome*					
Participation	Index zur Messung von Einschränkungen der Teilhabe (IMET) [19, 24]	●		●	●
*Secondary outcome*					
Psychological distress n	Center for Epidemiological Studies-Depression (CES-D) [27]	●	●	●	●
General health complaints	Symptom Checklist 90 Revised (SCL-90-R) [28]	●	●	●	●
Subjective health–related quality of life	World Health Organization Quality of Life-BREF (WHOQUOL-BREF) [29, 30]	●	●	●	●
Social support	Fragebogen zur sozialen Unterstützung (F – SozU) [31]	●		●	●
Performance in different areas of life	Single scales [32, 33]	●		●	●
Support offers	Single items	●		●	●
*Moderator variables*					
Height, weight, smoking	Single items [34]	●		●	●
Socio-demographic data	Deck & Röckelein 1999 [35]	●		●	●

#### Primary outcome

Participation restrictions are recorded using the index for the measurement of restrictions on participation (Index zur Messung von Einschränkungen der Teilhabe, IMET [19, 24]). This determines, using ICF criteria [25], the subjective impairment of chronically ill people in their everyday lives. It captures restrictions on participation in nine areas relevant to everyday life on a scale ranging from 0 to 10 and can be evaluated both at the individual item level and as a total score. High values indicate high participation restrictions. The IMET has proven to be valid and reliable for application in chronically ill patients [19, 26].

#### Secondary outcomes

Depressed mood states are measured with the German version of CES-D, which contains 20 quadruple scaled items. The presence and frequency of depressive symptoms are thus tested for. The evaluation is performed through a sum score, which can reach a maximum value of 60 points. A higher value describes a stronger expression of depressive symptoms. Good reliability and validity are reported [27].

For the general complaints, the subscale of somatization from the Symptom Checklist by Franke (SCL-90R) is used. This scale captures subjective impairment due to physical complaints via 12 five-fold stepped items. High values indicate high impairments. The reliability and validity of the scale have been proven [28].

The WHOQOL-BREF is used to assess the generic subjective perceived quality of life of the family carers. It contains 26 items on four dimensions; high values indicate a high quality of life. It is a valid and reliable cross-indication measuring instrument for assessing the quality of life [29, 30].

The extent of social support is assessed with the questionnaire for social support (FSozU K22). It contains 22 items; high values indicate a high level of social support. High reliability and validity have been proven [31].

Performance in the areas of life, everyday life, leisure and job, are captured on three numerical scales ranging from 0 to 10, with high values indicating high performance. In routine data collection, the three items have been proven to be a valid method of determining performance limitations [32, 33].

The utilization of support services will be recorded by single items used in a recent study on the rehab of carers. There will be standardized queried, inter alia, nursing service, self-help groups, household help, pastoral care and use and type of other offerings.

Moderator variables, risk factors [34] and socio-demographic variables [35] will also be recorded.

### Process evaluation

Items for process control, feasibility and satisfaction address the feasibility and acceptance of the intervention strategy by rehabilitants and staff. For this purpose, different single items (five-tier Likert scales) are used, which were developed and used in comparable studies [18].

In terms of the process control, the short structured evaluation forms completed by the participants after each telephone-based aftercare group are first sent to the moderator, who can use this information to form the next aftercare group session. For the evaluation of the process quality over the course of the intervention, the structured evaluation forms and all documentation records of each telephone-based aftercare group session (immediate and memory protocols) are evaluated in the ISE after the end of intervention. The experiences of the family carers with the telephone-based aftercare groups are requested during interviews at the end of the intervention and 6 months after the end of the intervention (*N* = 16). The focus here is particularly on the personal benefits and the temporal feasibility of the telephone-based aftercare groups, the satisfaction with these groups and the group composition. In addition, the wishes and needs of the participants or carers, respectively, are recorded, with regard to the further development or optimization of the telephone-based aftercare groups. To ensure the most heterogeneous sample composition, participants with different characteristics of the variables gender, education and time load in hours/day for the care of the family member with dementia are recruited for the interviews [36]. In addition, there will be a focus group discussion with the participating moderators of the telephone-based aftercare groups at the end of the intervention. Here, the feasibility aspects and the possibilities for further development will be focused on.

### Health economic evaluation

As part of the health economic evaluation, a cost-benefit analysis is performed. The EQ-5D 3 L is used to determine the values in use [37]. To calculate the index, the tariff, according to Greiner et al., is used as a guide [38]. When recording the costs, it is assumed that the costs of rehab are the same in both groups and, thus, only the costs of the intervention are relevant. In the health economic evaluation, therefore, the additional costs for the intervention are compared with the possible improvements in the utility values.

### Data management

#### Documentation in the rehab facility

For the organization and documentation of the flow of participants in the participating rehab facility, an automated documentation (Excel file) will be created. Staff responsible for recruitment in the rehab facility will be trained to use this file before the beginning of the study. For the consecutive numbering of enclosed subjects, each participant will be assigned a three-digit identification number (ID) (pseudonymization of data). All personal data will be documented on a separate data sheet in the Excel file; a corresponding link is made via the ID. Before transferring the data to the ISE, the personal information will be deleted. The collection of these data for standardized documentation is carried out exclusively in the participating rehab facility. Data generated by means of questionnaires will be collected and stored separately.

#### Data entry

Questionnaire data will be entered by a student assistant under the guidance of a research associate. Once the data have been entered, they will be checked for validity and plausibility by random double entries (5–10%).

### Data analysis

#### Effect analysis

For the effects analysis of the longitudinal data, analysis of variance with repeated measurements is used; for nominal and ordinal scaled parameters, contingency table analyses are used; and for subgroup analyses, chi-square tests and t-tests are used. The analyses are performed with SPSS 22.0 and complete cases will be analysed.

#### Process analysis

The short structured evaluation forms for the telephone-based aftercare groups are evaluated descriptively, and frequencies and proportions, mean values and scattering measures are reported. The focus of the evaluation is the identification of problem areas, which can then be reduced in future projects. The interviews are evaluated through structuring qualitative content analysis [39, 40]. The evaluations are computer-aided and performed with the program MAXQDA 12. To systematically describe relevant topics in the form of a category system, all transcripts are theory-guided and worked through with regard to interesting content-related aspects. The main topics (upper categories) are derived deductively from the research questions and the interview guide; subcategories are developed inductively from the material, for example, by subsumption [36]. For verification of the category system, an independent test coding of part of the material, with possibly subsequent modification of the categories and their definitions, is performed by two scientists. The entire coding process is performed in the form of consensual coding [40], viz. the transcripts are initially coded independently by two scientists. Afterwards, both codings are merged, and a consensus is developed.

#### Health economic analysis

Within the cost-utility-value analysis for the utility value, which is collected at three measurement times, the area under the curve (AUC) is calculated separately for IG and CG. The difference in the AUC values between IG and CG describes the difference in the quality-adjusted years of life. This difference is contrasted to the difference in costs.

### Study Progress

The study design and protocol were approved by the Ethics Committee of the University of Lübeck on 5 April 2018 (Register number: 18–059). The recruitment and collection of informed consent started in June 2018. The written survey of baseline data for the first participants also started in June 2018. The last T_3_ measurements, effect and process evaluation, are scheduled for July 2020. Analysis of the data, dissemination and publication of results are planned for the second half of the year 2020 and the first quarter of 2021.

## Discussion

Within the scope of the present project, for the first time, specifically for family carers of people with dementia, a developed support program will be tethered to a promising and, thus far, unique inpatient rehab program for this target group. The innovative content of this aftercare program concerns both the target group of family carers and the implementation method, in the form of telephone-based aftercare groups. For carers, the exchange with others is extremely important: they are experts in the care of their relatives with dementia; thus, they can support one another and provide valuable advice. Therefore, the participants in group interventions experience an emotionally relieving and supportive opportunity to have personal exchanges with other people who are in a comparable stressful situation [41, 42].

However, such offerings are often difficult to deliver to carers. Organizing the care of the person with dementia and the poor accessibility of locally attached support groups, particularly in rural areas, are obstacles to participating in a group away from their own homes [43–45]. Internationally, some so-called remote interventions have been developed for carers of people with dementia in recent years. These programs provide social support through online networks, chat forums, videophone or telephone, thereby overcoming location dependence. Overall, remote social support interventions have delivered promising results [46]. In a pilot study in Germany, a web-based psychoeducational training program for family carers of people with dementia was conducted and evaluated. Here, diverse barriers to the utilization of internet technology by carers have been disclosed (e.g., a still low pervasiveness of an internet broadband network in rural areas, fears of carers about computer technology and of the connection procedures of such computer-aided interventions in general) [47]. In Germany, the lead author of the present study tested structured telephone-based support groups for carers of people with dementia [16], according to the format developed for the REACH II support program [48]. Further attempts in this direction are not yet known in Germany. Based on the findings of Jonas and Trossmann [47] and results of Berwig and colleagues [49], the Talking Time – REHAB intervention is performed by telephone. Telephone-based services have great advantages: they can be performed regardless of location, and telephones are available in most households in Germany. In contrast to internet-based offers, in telephone-based offers, no utilization barriers from elderly people are expected.

In the present project, using telephone-based aftercare groups of Talking Time – REHAB intervention, a location-independent exchange and support possibility for family carers, is to be directly attached to an in-patient treatment for the first time and thereby integrated into a longer care process. It can be expected that the promising results of the remote social support offerings described above can even be exceeded.

Upon successful evaluation, the offer to participate in telephone-based aftercare groups would be able to be firmly established with a relatively small personal additional expense at the participating rehabilitation clinic. However, through minor adjustments, the offer would also be suitable for caregiving relatives of physically ill people. In addition, the principle of telephone-based aftercare in groups can also be used and adjusted for other, non-nursing-specific rehab indications.

## Abbreviations

AUC: Area under the curve
CES-D: Center for epidemiological studies–depression
CG: Control group
EQ-5D 3 L: EuroQol – 5 Dimensions 3 Levels
ES: Effect size
F-SozU: Fragebogen zur sozialen Unterstützung (Questionnaire on Social Support)
IG: Intervention group
IMET: Index zur Messung von Einschränkungen der Teilhabe (Measurement of Restrictions on Participation)
ISE: Institute for Social Medicine and Epidemiology
MAXQDA: MAX qualitative data analysis
N: Number
Rehab: Rehabilitation
SCL-90-R: Symptom Checklist 90 Revised
SGB: Sozialgesetzbuch
SPSS: Statistical package for the social science
TCI: Theme-centred interaction
WHOQUOL-BREF: World Health Organization Quality of Life–BREF Instrument
